# 2-Chloro-*N*-(4-sulfamoylphen­yl)acetamide

**DOI:** 10.1107/S1600536810021185

**Published:** 2010-06-05

**Authors:** Mehmet Akkurt, Şerife Pınar Yalçın, Hasan Türkmen, Orhan Büyükgüngör

**Affiliations:** a Department of Physics, Faculty of Sciences, Erciyes University, 38039 Kayseri, Turkey; b Department of Physics, Faculty of Arts and Sciences, Harran University, 63300 Şanlıurfa, Turkey; c Department of Chemistry, Faculty of Arts and Sciences, Harran University, 63300 Şanlıurfa, Turkey; d Department of Physics, Faculty of Arts and Sciences, Ondokuz Mayıs University, 55139 Samsun, Turkey

## Abstract

In the title compound, C_8_H_9_ClN_2_O_3_S, the benzene ring makes a dihedral angle of 4.1 (9)° with the amido –NHCO– plane including the major occupancy component of the carbonyl O atom [19 (4)° for the minor component]. An intra­molecular C—H⋯O inter­action occurs. The O atom of the carbonyl group is disordered over two positions with site-occupancy factors of 0.67 (11) and 0.33 (11). Inter­molecular N—H⋯O hydrogen bonds help to stabilize the crystal structure.

## Related literature

For the anti­bacterial activity of sulfonamides and their derivatives and for their pharmacological applications, see: Köhler *et al.* (2007 ▶); Ohradanova *et al.* (2007 ▶); Supuran (2008 ▶); Türkmen *et al.* (2005 ▶); Thiry *et al.* (2008 ▶). For comparative bond lengths, see: Allen *et al.* (1987 ▶).
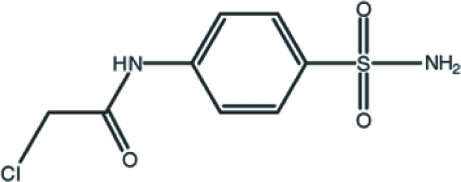


## Experimental

### 

#### Crystal data

C_8_H_9_ClN_2_O_3_S
*M* *_r_* = 248.69
Monoclinic, 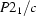

*a* = 4.7870 (2) Å
*b* = 14.1868 (9) Å
*c* = 16.0158 (9) Å
β = 90.907 (4)°
*V* = 1087.53 (10) Å^3^
*Z* = 4
Mo *K*α radiation
μ = 0.53 mm^−1^
*T* = 296 K
0.72 × 0.50 × 0.35 mm

#### Data collection

Stoe IPDS2 diffractometer
Absorption correction: integration (*X-RED32*; Stoe & Cie, 2002 ▶) *T*_min_ = 0.734, *T*_max_ = 0.830
12474 measured reflections
2193 independent reflections
2021 reflections with *I* > 2σ(*I*)
*R*_int_ = 0.032

#### Refinement

*R*[*F* ^2^ > 2σ(*F* ^2^)] = 0.050
*wR*(*F* ^2^) = 0.129
*S* = 1.05
2193 reflections
154 parameters
2 restraints
H atoms treated by a mixture of independent and constrained refinement
Δρ_max_ = 0.56 e Å^−3^
Δρ_min_ = −0.53 e Å^−3^

### 

Data collection: *X-AREA* (Stoe & Cie, 2002 ▶); cell refinement: *X-AREA*; data reduction: *X-RED32* (Stoe & Cie, 2002 ▶); program(s) used to solve structure: *SIR97* (Altomare *et al.*, 1999 ▶); program(s) used to refine structure: *SHELXL97* (Sheldrick, 2008 ▶); molecular graphics: *ORTEP-3* (Farrugia, 1997 ▶); software used to prepare material for publication: *WinGX* (Farrugia, 1999 ▶).

## Supplementary Material

Crystal structure: contains datablocks global, I. DOI: 10.1107/S1600536810021185/xu2770sup1.cif

Structure factors: contains datablocks I. DOI: 10.1107/S1600536810021185/xu2770Isup2.hkl

Additional supplementary materials:  crystallographic information; 3D view; checkCIF report

## Figures and Tables

**Table 1:** Hydrogen-bond geometry (Å, °)

*D*—H⋯*A*	*D*—H	H⋯*A*	*D*⋯*A*	*D*—H⋯*A*
N1—H1*A*⋯O1^i^	0.85 (2)	2.12 (2)	2.886 (3)	151 (3)
N1—H1*B*⋯O3*B*^ii^	0.83 (3)	2.15 (4)	2.95 (3)	162 (4)
N2—H2*A*⋯O2^iii^	0.86	2.14	3.002 (3)	175
C5—H5⋯O3*B*	0.93	2.23	2.84 (2)	122

Symmetry codes: (i) 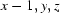; (ii) 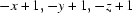; (iii) 
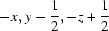
.

**Fig. 1.**
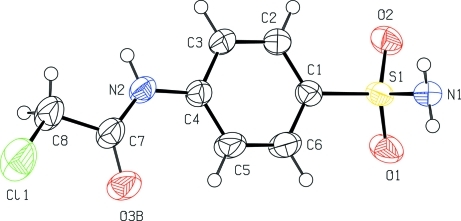
The molecular structure of (I) with 50% probability ellipsoids for non-H atoms. Only the major disordered component is shown.

**Fig. 2.**
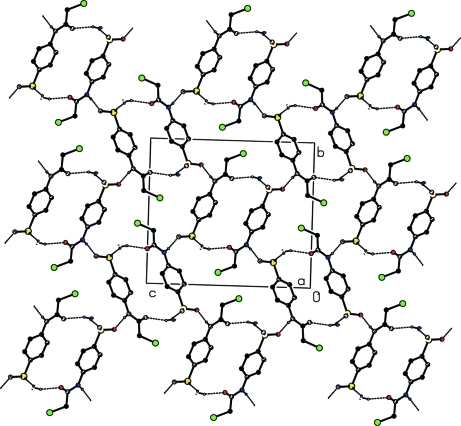
The crystal packing and hydrogen bonding interactions of (I), viewed down the a axis. All hydrogen atoms not involved in hydrogen bonding and the minor disorder component have been omitted for clarity.

## supplementary crystallographic information

### Comment

Sulfonamide is the basis of several groups of drugs. The original antibacterial sulfonamides (sometimes called simply sulfa drugs) are synthetic antimicrobial agents that contain the sulfonamide group. Some sulfonamides are also devoid of antibacterial activity, *e.g.*, the anticonvulsant sultiame. The sulfonylureas and thiazide diuretics are newer drug groups based on the antibacterial sulfonamides. Sulfanilamide is a sulfonamide antibacterial.

Carbonic anhydrases (CAs, EC 4.2.1.1) are widespread metalloenzymes in bacteria, archaea, and eukaryotes, catalyzing a critically important physiologic reaction, hydration of carbon dioxide to bicarbonate and protons (Ohradanova *et al.*, 2007; Supuran, 2008). These enzymes are inhibited by several classes of compounds, such as sulfonamides, sulfamates and sulfamides some of which have pharmacologic applications for the treatment of glaucoma obesity cancer epilepsy and other neurological disorders or as diuretics (Supuran, 2008; Köhler *et al.*, 2007; Türkmen *et al.*, 2005; Thiry *et al.*, 2008). In view of these importance, we have undertaken the crystal structure determination of the compound *2-chloro-N-(4-sulfamoylphenyl)acetamide* and the results are presented here.

In the molecular structure of the title compound, (I), (Fig. 1), the S=O distances are 1.432 (2) and 1.433 (2) Å, and the angle of O=S=O is 118.38 (14)°. All the bond lengths and the bond angles are within the normal range (Allen *et al.*, 1987). The planes of the benzene ring and the O=S=O group make a dihedral angle of 126.81 (11)°. The C4—N2—C7—C8, N2—C7—C8—Cl1 and C4—N2—C7—O3B torsion angles in the 2-chloroacetamide part of the molecule are -173.3 (3), -157.8 (2) and -4.8 (14)°, respectively.

In the crystal structure, symmetry-related molecules are interconected by intermolecular N—H···O hydrogen bonds (Table 1) to form a three-dimensional network (Fig.2).

### Experimental

Sulfanilamide (1.00 g, 0.0058 mol) and *N*-ethylmaleimide (NEM) (0.80 g, 0.007 mol) were stirred in THF (200 ml) until most of the starting material had dissolved. 2-Chloroethanoylchloride (0.784 g, 0.007 mol) in THF was slowly added to the reaction mixture. The reaction was stirred at 258 K for 4 h under anhydrous conditions. After warming to room temperature the white precipitate of NEM/HCl salt filtered off. The THF was removed in *vacuo* and the resulting white solid dissolved in ethyl acetate. The organic extract was washed with 3*M* hydrochloric acid (20 ml) then with saturated sodium bicarbonate solution (20 ml) and finally with brine. Drying over magnesium sulfate and evaporation yielded a white solid which was recrystallized from water to give the title compound (yield: 70%, m.p: 492–495 K).

### Refinement

The NH_2_ H atoms were located in a difference Fourier map, and were refined with distance restraints of N–H = 0.86 (2) Å; their temperature factors were freely refined. The rest H atoms were positioned geometrically and allowed to ride on their respective parent atoms, with C—H = 0.93 (Ar—H) or 0.97 (CH_2_) Å and N—H = 0.86 (NH) \%A, and with *U*_eq_ = 1.2*U*_eq_(C, N). The O atom of the carbonyl group is disorder over two sets of sites [occupancy ratio = 0.67 (11):0.33 (11)].

### Crystal data

**Table:**

C_8_H_9_ClN_2_O_3_S	*F*(000) = 512
*M**_r_* = 248.69	*D*_x_ = 1.519 Mg m^−^^3^
Monoclinic, *P*2_1_/*c*	Mo *K*α radiation, λ = 0.71073 Å
Hall symbol: -P 2ybc	Cell parameters from 27592 reflections
*a* = 4.7870 (2) Å	θ = 1.9–28.1°
*b* = 14.1868 (9) Å	µ = 0.53 mm^−^^1^
*c* = 16.0158 (9) Å	*T* = 296 K
β = 90.907 (4)°	Prism, colourless
*V* = 1087.53 (10) Å^3^	0.72 × 0.50 × 0.35 mm
*Z* = 4	

### Data collection

**Table:**

Stoe IPDS2 diffractometer	2193 independent reflections
Radiation source: sealed X-ray tube, 12 x 0.4 mm long-fine focus	2021 reflections with *I* > 2σ(*I*)
plane graphite	*R*_int_ = 0.032
Detector resolution: 6.67 pixels mm^-1^	θ_max_ = 26.5°, θ_min_ = 1.9°
ω scans	*h* = −6→5
Absorption correction: integration (*X-RED32*; Stoe & Cie, 2002)	*k* = −17→17
*T*_min_ = 0.734, *T*_max_ = 0.830	*l* = −20→20
12474 measured reflections	

### Refinement

**Table:**

Refinement on *F*^2^	Primary atom site location: structure-invariant direct methods
Least-squares matrix: full	Secondary atom site location: difference Fourier map
*R*[*F*^2^ > 2σ(*F*^2^)] = 0.050	Hydrogen site location: inferred from neighbouring sites
*wR*(*F*^2^) = 0.129	H atoms treated by a mixture of independent and constrained refinement
*S* = 1.05	*w* = 1/[σ^2^(*F*_o_^2^) + (0.0576*P*)^2^ + 1.1572*P*] where *P* = (*F*_o_^2^ + 2*F*_c_^2^)/3
2193 reflections	(Δ/σ)_max_ < 0.001
154 parameters	Δρ_max_ = 0.56 e Å^−^^3^
2 restraints	Δρ_min_ = −0.53 e Å^−^^3^

### Special details

**Table:**

Geometry. Bond distances, angles *etc*. have been calculated using the rounded fractional coordinates. All su's are estimated from the variances of the (full) variance-covariance matrix. The cell e.s.d.'s are taken into account in the estimation of distances, angles and torsion angles
Refinement. Refinement on *F*^2^ for ALL reflections except those flagged by the user for potential systematic errors. Weighted *R*-factors *wR* and all goodnesses of fit *S* are based on *F*^2^, conventional *R*-factors *R* are based on *F*, with *F* set to zero for negative *F*^2^. The observed criterion of *F*^2^ > σ(*F*^2^) is used only for calculating -*R*-factor-obs *etc*. and is not relevant to the choice of reflections for refinement. *R*-factors based on *F*^2^ are statistically about twice as large as those based on *F*, and *R*-factors based on ALL data will be even larger.

### Fractional atomic coordinates and isotropic or equivalent isotropic displacement parameters (Å^2^)

**Table:**

	*x*	*y*	*z*	*U*_iso_*/*U*_eq_	Occ. (<1)
Cl1	0.2379 (3)	0.08886 (7)	0.56592 (6)	0.0959 (5)	
S1	0.41100 (13)	0.66791 (5)	0.26923 (4)	0.0393 (2)	
O1	0.7050 (4)	0.68487 (16)	0.27785 (16)	0.0597 (7)	
O2	0.2847 (4)	0.67525 (14)	0.18777 (12)	0.0495 (6)	
O3B	0.500 (4)	0.2630 (11)	0.5011 (18)	0.065 (3)	0.67 (11)
N1	0.2607 (5)	0.74276 (17)	0.32797 (15)	0.0452 (7)	
N2	0.1740 (5)	0.28448 (15)	0.39800 (13)	0.0435 (7)	
C1	0.3465 (5)	0.55335 (18)	0.30663 (15)	0.0386 (7)	
C2	0.1432 (6)	0.4980 (2)	0.26979 (17)	0.0488 (9)	
C3	0.0903 (7)	0.40929 (19)	0.30111 (19)	0.0515 (9)	
C4	0.2414 (5)	0.37552 (18)	0.36939 (15)	0.0392 (7)	
C5	0.4491 (7)	0.4307 (2)	0.4052 (2)	0.0587 (10)	
C6	0.5012 (7)	0.5193 (2)	0.3732 (2)	0.0593 (10)	
C7	0.2930 (7)	0.2361 (2)	0.46056 (18)	0.0534 (10)	
C8	0.1863 (9)	0.1357 (2)	0.4664 (2)	0.0679 (13)	
O3A	0.40 (2)	0.277 (3)	0.522 (3)	0.078 (14)	0.33 (11)
H1A	0.087 (4)	0.733 (2)	0.331 (2)	0.050 (9)*	
H1B	0.342 (7)	0.753 (3)	0.3734 (15)	0.068 (11)*	
H2A	0.03930	0.25660	0.37180	0.0520*	
H3	−0.04750	0.37190	0.27630	0.0620*	
H5	0.55320	0.40830	0.45050	0.0700*	
H6	0.64200	0.55620	0.39690	0.0710*	
H8A	0.28220	0.09680	0.42620	0.0810*	
H8B	−0.01160	0.13450	0.45240	0.0810*	
H2	0.04170	0.52030	0.22390	0.0590*	

### Atomic displacement parameters (Å^2^)

**Table:**

	*U*^11^	*U*^22^	*U*^33^	*U*^12^	*U*^13^	*U*^23^
Cl1	0.1553 (12)	0.0675 (6)	0.0641 (6)	−0.0342 (7)	−0.0249 (6)	0.0246 (5)
S1	0.0328 (3)	0.0429 (4)	0.0419 (4)	−0.0018 (2)	−0.0056 (2)	0.0102 (3)
O1	0.0346 (10)	0.0627 (13)	0.0816 (15)	−0.0044 (9)	−0.0056 (10)	0.0247 (11)
O2	0.0540 (11)	0.0552 (12)	0.0390 (10)	0.0032 (9)	−0.0051 (8)	0.0106 (8)
O3B	0.083 (6)	0.062 (3)	0.050 (5)	−0.022 (4)	−0.027 (4)	0.013 (3)
N1	0.0438 (13)	0.0435 (12)	0.0480 (13)	−0.0004 (10)	−0.0117 (11)	−0.0003 (10)
N2	0.0545 (13)	0.0377 (11)	0.0378 (11)	−0.0089 (10)	−0.0099 (9)	−0.0018 (9)
C1	0.0396 (13)	0.0397 (13)	0.0364 (12)	−0.0002 (10)	−0.0028 (10)	0.0032 (10)
C2	0.0604 (17)	0.0414 (14)	0.0439 (14)	0.0004 (12)	−0.0188 (12)	−0.0006 (11)
C3	0.0627 (17)	0.0386 (14)	0.0524 (16)	−0.0055 (12)	−0.0239 (13)	−0.0039 (12)
C4	0.0463 (14)	0.0371 (12)	0.0342 (12)	−0.0022 (10)	−0.0034 (10)	−0.0012 (10)
C5	0.0675 (19)	0.0544 (17)	0.0532 (16)	−0.0206 (15)	−0.0274 (15)	0.0182 (14)
C6	0.0618 (18)	0.0562 (17)	0.0589 (18)	−0.0227 (15)	−0.0260 (15)	0.0172 (14)
C7	0.076 (2)	0.0426 (15)	0.0410 (14)	−0.0153 (14)	−0.0135 (14)	0.0035 (12)
C8	0.105 (3)	0.0449 (16)	0.0530 (17)	−0.0217 (17)	−0.0238 (18)	0.0112 (14)
O3A	0.14 (4)	0.050 (9)	0.042 (11)	−0.043 (14)	−0.044 (16)	0.017 (7)

### Geometric parameters (Å, °)

**Table:**

Cl1—C8	1.741 (3)	C1—C6	1.376 (4)
S1—O1	1.432 (2)	C2—C3	1.380 (4)
S1—O2	1.433 (2)	C3—C4	1.387 (4)
S1—N1	1.597 (2)	C4—C5	1.383 (4)
S1—C1	1.761 (3)	C5—C6	1.382 (4)
O3A—C7	1.25 (6)	C7—C8	1.517 (4)
O3B—C7	1.24 (2)	C2—H2	0.9300
N2—C4	1.410 (3)	C3—H3	0.9300
N2—C7	1.335 (4)	C5—H5	0.9300
N1—H1A	0.85 (2)	C6—H6	0.9300
N1—H1B	0.83 (3)	C8—H8A	0.9700
N2—H2A	0.8600	C8—H8B	0.9700
C1—C2	1.376 (4)		
			
Cl1···O3B	2.966 (18)	N2···O2^viii^	3.002 (3)
Cl1···O3A	2.87 (5)	N1···H3^v^	2.6700
Cl1···C2^i^	3.526 (3)	C2···O1^ii^	3.384 (4)
Cl1···H2^i^	3.1200	C2···C6^ii^	3.527 (4)
S1···O1^ii^	3.393 (2)	C2···Cl1^ix^	3.526 (3)
O1···S1^iii^	3.393 (2)	C3···C6^ii^	3.439 (5)
O1···O2^iii^	3.151 (3)	C3···C5^ii^	3.529 (5)
O1···N1^iii^	2.886 (3)	C5···O3B	2.84 (2)
O1···C2^iii^	3.384 (4)	C5···O3A	2.89 (5)
O1···N2^iv^	3.212 (3)	C5···C3^iii^	3.529 (5)
O2···C7^iv^	3.260 (4)	C6···O3A^vi^	3.37 (5)
O2···C8^v^	3.364 (4)	C6···C3^iii^	3.439 (5)
O2···N2^v^	3.002 (3)	C6···C2^iii^	3.527 (4)
O2···O1^ii^	3.151 (3)	C7···O2^vii^	3.260 (4)
O3A···C5	2.89 (5)	C8···O2^viii^	3.364 (4)
O3A···Cl1	2.87 (5)	C6···H5^vi^	3.0200
O3A···C6^vi^	3.37 (4)	C7···H5	2.7500
O3A···N1^vi^	2.89 (7)	H1A···O1^ii^	2.12 (2)
O3B···C5	2.84 (2)	H1B···O3B^vi^	2.15 (4)
O3B···Cl1	2.966 (18)	H1B···O3A^vi^	2.11 (7)
O3B···N1^vi^	2.95 (3)	H2···O2	2.5600
O1···H6	2.6600	H2···Cl1^ix^	3.1200
O1···H2A^iv^	2.8900	H2A···H8B	2.1800
O1···H1A^iii^	2.12 (2)	H2A···H3	2.2700
O2···H2A^v^	2.1400	H2A···O1^vii^	2.8900
O2···H2	2.5600	H2A···O2^viii^	2.1400
O2···H8B^v^	2.6400	H3···N1^viii^	2.6700
O3A···H5	2.3100	H3···H2A	2.2700
O3A···H6^vi^	2.7100	H5···O3B	2.2300
O3A···H1B^vi^	2.11 (7)	H5···C7	2.7500
O3B···H1B^vi^	2.15 (4)	H5···O3A	2.3100
O3B···H5	2.2300	H5···C6^vi^	3.0200
N1···O1^ii^	2.886 (3)	H6···O1	2.6600
N1···O3B^vi^	2.95 (3)	H6···O3A^vi^	2.7100
N1···O3A^vi^	2.89 (7)	H8B···H2A	2.1800
N2···O1^vii^	3.212 (3)	H8B···O2^viii^	2.6400
			
O1—S1—O2	118.38 (14)	C1—C6—C5	120.6 (3)
O1—S1—N1	106.42 (13)	O3A—C7—C8	122 (2)
O1—S1—C1	107.43 (13)	O3B—C7—C8	121.7 (9)
O2—S1—N1	107.48 (12)	O3B—C7—N2	124.4 (10)
O2—S1—C1	107.64 (12)	N2—C7—C8	112.9 (3)
N1—S1—C1	109.28 (12)	O3A—C7—N2	121 (2)
C4—N2—C7	128.2 (2)	Cl1—C8—C7	111.8 (2)
H1A—N1—H1B	115 (3)	C1—C2—H2	120.00
S1—N1—H1A	112 (2)	C3—C2—H2	120.00
S1—N1—H1B	115 (3)	C2—C3—H3	120.00
C4—N2—H2A	116.00	C4—C3—H3	120.00
C7—N2—H2A	116.00	C4—C5—H5	120.00
S1—C1—C2	120.5 (2)	C6—C5—H5	120.00
S1—C1—C6	119.5 (2)	C1—C6—H6	120.00
C2—C1—C6	120.0 (2)	C5—C6—H6	120.00
C1—C2—C3	119.8 (3)	Cl1—C8—H8A	109.00
C2—C3—C4	120.4 (3)	Cl1—C8—H8B	109.00
C3—C4—C5	119.6 (3)	C7—C8—H8A	109.00
N2—C4—C5	123.4 (2)	C7—C8—H8B	109.00
N2—C4—C3	117.0 (2)	H8A—C8—H8B	108.00
C4—C5—C6	119.6 (3)		
			
O1—S1—C1—C2	−144.9 (2)	S1—C1—C6—C5	178.1 (2)
O2—S1—C1—C2	−16.4 (2)	C2—C1—C6—C5	−1.9 (4)
N1—S1—C1—C2	100.1 (2)	C6—C1—C2—C3	1.6 (4)
O1—S1—C1—C6	35.1 (3)	C1—C2—C3—C4	−0.2 (4)
O2—S1—C1—C6	163.6 (2)	C2—C3—C4—N2	179.6 (3)
N1—S1—C1—C6	−79.9 (2)	C2—C3—C4—C5	−1.1 (4)
C7—N2—C4—C3	178.2 (3)	N2—C4—C5—C6	−179.9 (3)
C7—N2—C4—C5	−1.1 (4)	C3—C4—C5—C6	0.8 (4)
C4—N2—C7—O3B	−4.8 (14)	C4—C5—C6—C1	0.6 (5)
C4—N2—C7—C8	−173.3 (3)	O3B—C7—C8—Cl1	33.4 (14)
S1—C1—C2—C3	−178.4 (2)	N2—C7—C8—Cl1	−157.8 (2)

Symmetry codes: (i) *x*, −*y*+1/2, *z*+1/2; (ii) *x*−1, *y*, *z*; (iii) *x*+1, *y*, *z*; (iv) −*x*+1, *y*+1/2, −*z*+1/2; (v) −*x*, *y*+1/2, −*z*+1/2; (vi) −*x*+1, −*y*+1, −*z*+1; (vii) −*x*+1, *y*−1/2, −*z*+1/2; (viii) −*x*, *y*−1/2, −*z*+1/2; (ix) *x*, −*y*+1/2, *z*−1/2.

### Hydrogen-bond geometry (Å, °)

**Table:**

*D*—H···*A*	*D*—H	H···*A*	*D*···*A*	*D*—H···*A*
N1—H1A···O1^ii^	0.85 (2)	2.12 (2)	2.886 (3)	151 (3)
N1—H1B···O3B^vi^	0.83 (3)	2.15 (4)	2.95 (3)	162 (4)
N2—H2A···O2^viii^	0.86	2.14	3.002 (3)	175
C2—H2···O2	0.93	2.56	2.922 (3)	104
C5—H5···O3B	0.93	2.23	2.84 (2)	122

Symmetry codes: (ii) *x*−1, *y*, *z*; (vi) −*x*+1, −*y*+1, −*z*+1; (viii) −*x*, *y*−1/2, −*z*+1/2.
